# Prevention of Alzheimer Pathology by Blocking Neuregulin Signaling on Microglia

**DOI:** 10.1523/ENEURO.0422-23.2023

**Published:** 2023-11-08

**Authors:** Jianguo Liu, Joseph R. Geraghty, Sarah Schram, Haley C. Cropper, Justin Lei, Jeffrey A. Loeb, Fei Song

**Affiliations:** Department of Neurology and Rehabilitation, The University of Illinois at Chicago, Chicago, Illinois 60612

**Keywords:** microglia, neuregulin antagonist, neuregulin-1, neurodegeneration, neuroinflammation, therapeutic target

## Abstract

Plaque formation, microglial activation, and synaptic loss are pathologic hallmarks of Alzheimer’s disease; however, removing plaques has had little clinical benefit. Here, we show that neuregulin-1, a glial growth factor, induces inflammatory cytokines and promotes phagocytic activity *in vitro* and augments microglial activation and plaque formation in 5XFAD Alzheimer’s mice. Brain-specific targeting of neuregulin-1 by intraventricular delivery of a novel neuregulin-1 fusion protein antagonist, GlyB4, significantly alters microglial morphology and function to a nonpathogenic morphology in early-stage 5XFAD mice and prevents plaques from forming. Once plaques have already formed, GlyB4 reduces new plaque formation and prevents synaptic loss. Selective, targeted disruption of neuregulin-1 signaling on brain microglia with GlyB4 could be a novel “upstream” approach to slow or stop disease progression in Alzheimer’s disease.

## Significance Statement

Microglia-associated neuroinflammation is a major hallmark of Alzheimer’s disease. Here, we show that neuregulin1 (NRG1), an important neuron-glial signaling factor in development, induces microglial activation and promotes pathologic changes in the 5XFAD mouse model of Alzheimer’s disease. Inhibition of NRG1 using a targeted NRG1 antagonist called GlyB4 blocks NRG1 receptor activation in microglia, reduces amyloid β plaque formation, and prevents synaptic loss. While NRG1 clearly has important functions at other sites in the nervous system, excessive NRG1 signaling in microglia leads to pathologic neuroinflammation and neurodegeneration. Selective disruption of NRG1 signaling in microglia using GlyB4 could be a novel approach to block disease progression in patients with Alzheimer’s disease.

## Introduction

Alzheimer’s disease (AD) is progressive and characterized by memory impairments and cognitive decline (Schultz et al., 2004). AD is the sixth leading cause of death in the United States and has no effective treatments. Histopathologically, the AD brain contains amyloid β (Aβ) plaques, tau-containing neurofibrillary tangles, activated microglia, and extensive synaptic and neuronal loss (Dani et al., 2018; Leng and Edison, 2021). A concerted effort in the field has focused on treatments that remove plaques and tangles once they have formed; however, to date these therapeutic approaches have had minimal effects on retarding disease progression and disability (Ruthirakuhan et al., 2016). Upstream from plaque formation, neuroinflammation can drive AD progression leading to Aβ plaque and tau tangle deposition (Kinney et al., 2018; Pascoal et al., 2021). As the primary players in brain inflammation (Koenigsknecht and Landreth, 2004), microglia are brain-resident myeloid cells that can induce neuronal dysfunction and loss through mechanisms of increased phagocytosis, cytokine secretion, and synaptic pruning, and changes in trophic support (McQuade and Blurton-Jones, 2019). In fact, progressive decline of memory function and cognition in AD correlates best with neuronal and synaptic loss (Coleman et al., 2004; Brenner, 2005). These findings have led to a number of efforts to block microglial activation as a potentially more effective “upstream” therapeutic strategy.

Neuregulin 1 (NRG1) is a neuronally derived protein factor that has critical roles in glial growth and development (Gassmann et al., 1995; Lee et al., 1995; Meyer and Birchmeier, 1995). *NRG1* produces a number of isoforms that target it to different cells with different functions through alternative splicing. It can exist either as a soluble heparin-binding factor or as a membrane-bound form important for direct cell–cell interactions (Falls, 2003; Mei and Xiong, 2008; Pankonin et al., 2009; Ma et al., 2011; Wang et al., 2015). Its diverse signaling capabilities enable it to have highly localized effects in CNS and peripheral nervous system development regulating cell growth, survival, and differentiation of glial, muscle, cardiac, and neuronal cells (Falls, 2003; Mei and Xiong, 2008; Pankonin et al., 2009; Ma et al., 2011; Wang et al., 2015). In the mature nervous system, NRG1 has a different role in the setting of injury as a potent activator of microglia that can lead to synaptic changes contributing to chronic pain (Calvo et al., 2010, 2011) and neurodegeneration in amyotrophic lateral sclerosis (ALS; Song et al., 2012, 2014; Liu et al., 2018; Schram et al., 2020). Microglia express all three NRG1 receptors, erbB2, erbB3, and erbB4 (Gerecke et al., 2001; Dimayuga et al., 2003; Calvo et al., 2010, 2011), and NRG1 has been shown to be a survival, proliferative, and chemotactic factor for microglia *in vitro* (Calvo et al., 2011).

NRG1 is released into the CNS from injured peripheral sensory neurons, promotes pathologic neuroinflammation, and leads to the development of chronic pain (Lacroix-Fralish et al., 2008; Calvo et al., 2010). We have shown that several different NRG1 inhibitors, including intrathecal GlyB4, can block microglial activation in this chronic pain model (Calvo et al., 2010, 2011). Similarly, we found that intraventricular GlyB4 can block microglial activation and motor neuron loss in an animal model of ALS (Song et al., 2012, 2014; Liu et al., 2018). GlyB4 is a novel fusion protein containing a “decoy” extracellular HER4 NRG1 receptor fused to the heparin-binding domain of NRG1, which targets the decoy receptor to the same sites where NRG1 acts (Ma et al., 2009; Loeb and Song, 2019), leading to reduced microgliosis and synapse and motor neuron loss in ALS animals (Liu et al., 2018).

NRG1 has recently been implicated in AD pathogenesis. In spinal fluid from mild cognitive impairment (MCI) AD patients, NRG1 levels are increased and associated with cognitive loss (Pankonin et al., 2009; Mouton-Liger et al., 2020; Vrillon et al., 2022). A single nucleotide polymorphism of the NRG1 gene (rs392499) is associated with late-onset AD (Go et al., 2005). Furthermore, BACE, the enzyme that generates Aβ from APP, also releases soluble NRG1 from its transmembrane precursor, further linking NRG1 to human genetic and postmortem studies (Mouton-Liger et al., 2020). Finally, the NRG1 receptors (erbB2–4) are altered in mouse models of AD (Chaudhury et al., 2003; Repetto et al., 2007; Zhang et al., 2007).

Given that NRG1 promotes neuroinflammation and that blocking NRG1 reduces neuroinflammation prevents neuronal loss in ALS, here we explored the effects of NRG1 and GlyB4 on early AD disease pathology in the 5XFAD animal model. We found that adding higher levels of NRG1 intraventricularly promotes AD pathology, while blocking NRG1 signaling with GlyB4 induces morphologic changes in microglia associated with reduced plaque formation and synaptic loss. While other studies suggest that NRG1 signaling at other sites could have beneficial roles in AD (Ryu et al., 2016; Xu et al., 2016; Ou et al., 2021), here we show that specifically targeting NRG1 signaling on CNS microglia can slow or stop neuroinflammation and neurodegeneration in AD animals.

## Materials and Methods

### Mice

CX_3_CR-1^GFP^ (B6.129P2(Cg)-*Cx3cr1^tm1Litt^*/J; strain #005582) were purchased from The Jackson Laboratory for *in vitro* microglial studies. 5XFAD (Familiar Alzheimer Disease) transgenic (Tg) mice are an aggressive model of AD-related pathology with intracellular amyloid β and amyloid β plaques appearing that was accompanied by gliosis as early as 2 months (Oakley et al., 2006). Six-week-old male and female 5XFAD mice [B6.Cg Tg (APPSwFlLon,PSEN1*M146L*L286V)6799Vas/Mmjax; strain MMRRC #034848-JAX] were purchased from The Jackson Laboratory under a Material Transfer Agreement. 5XFAD mice were maintained individually in a specifically designed mouse cage to prevent accidental removal of intracerebroventricular catheters after brain surgery at the Animal Facility of the University of Illinois at Chicago. All procedures including animal experiments were approved by the Animal Research Ethics Committee, University of Illinois at Chicago.

### Microglial isolation

Primary mixed mouse glial cell cultures were prepared based on protocol adapted from Tamashiro et al. (2012). Briefly, mouse brains were harvested and dissected from postnatal day 0 (P0) to P5 male and female CX3CR-1^GFP^ mice under a stereomicroscope. Brains were washed three times in cold HBSS (catalog #14175095, Thermo Fisher Scientific) and subsequently incubated with 0.25% trypsin (catalog #15400054, Thermo Fisher Scientific) and 50 μg of DNase I (catalog #FEREN0521, Thermo Fisher Scientific) for 7 min at room temperature. Ten milliliters of complete DMEM containing 4.5 g of glucose, l-glutamine, 110 mg/L sodium pyruvate, 10% fetal bovine serum (FBS; catalog #F2442, Sigma-Aldrich), and 1% antibiotic-antimycotic solution (catalog #15240062, Thermo Fisher Scientific) was added to quench the reaction, followed by wash with HBSS an additional two times before being passed five times through pipettes of decreasing diameter to promote tissue dissociation. The suspension was then filtered through a 70 μm nylon cell strainer (catalog #087712, Corning Falcon), spun at 300 g for 10 min, and subsequently plated in 15 ml of complete DMEM and cultured at 37°C with 5% CO_2_. After 24 h, media was replaced with 15 ml of fresh complete DMEM. Two-thirds of the media was replaced every 3–4 d until confluent. Approximately three to four brains were cultured per T-75 cm^2^ flask (catalog #1368065, Corning Falcon). Mixed glial cultures became confluent 12–14 d after plating (Tamashiro et al., 2012; Lian et al., 2016a). Microglia were then isolated from the mixed glial population by sealing the flasks and shaking at 300 rpm for 30 min at 37°C. The media containing microglia was collected and used for subsequent experiments. Isolated microglia were incubated at a density of 20,000 cells/mm^2^ in eight-well chamber slides (catalog #PEZGS0816, MilliporeSigma) precoated with 10 μg/ml poly-l-lysine at 37°C with 5% CO_2_ for 24 h.

### Flow cytometry

Isolated microglia (CX_3_CR-1^GFP+^ cells) and possibly remaining astrocytes (CX3CR1-gfp- cells) obtained from the isolation procedure above were resuspended in eBioscience Flow Cytometry Staining buffer (BD PharMingen) and immunostained with rat anti-mouse CD45-PE-Cy7 antibody (catalog #552848, BD PharMingen) for 30 min in the dark at 4°C. After washing, the cells were permeabilized and fixed (BD Cytofix/Cytoperm Solution, BD Biosciences) for 20 min in the dark at room temperature. The CD45 antibody was cy7 fluorochrome conjugated and used at a final concentration of 0.2 mg/ml. Finally, the cells were fixed with 1× eBioscience Permeabilization Buffer (catalog #561651, BD Biosciences) and stored at 4°C until analysis within 24 h by a LSRFortessa Cell Analyzer (BD Biosciences). Samples were analyzed with FlowJo software (Tree Star).

### Microglial culture and phagocytosis assay

Before all experiments, a 3 h incubation was performed with serum-free DMEM. Serum-free media was used for the remainder of the experiment given that serum exposure promotes a nonphysiological environment that can induce a reactive, amoeboid state in microglia (Guttenplan and Liddelow, 2019). Microglia were exposed to 0.1 or 10 nm neuregulin (NRG1-β 1/HRG1-β 1 ECD protein; catalog #377-HB, R&D Systems) diluted in a 0.1% bovine serum albumin (BSA) solution (catalog #A2153, Sigma-Aldrich). Lipopolysaccharide (L6529, Sigma-Aldrich, Inc., St. Louis, Missouri) 4 μg/ml was used as a positive control.

The phagocytic activity of primary CX_3_CR1^GFP^ microglia cells in response to NRG1 and LPS was assayed using a fluorescent latex bead phagocytosis assay (Lian et al., 2016b). Two-micrometer fluorescent blue, amine-modified polystyrene latex beads in aqueous suspension were purchased from Sigma-Aldrich (catalog #L0280). Latex beads were first incubated in a 1:5 dilution of heat-inactivated FBS before experimentation. Final bead concentration applied to cells was 0.02% (1:5000 dilution). Cells were incubated for 12 h at 37°C in 5% CO_2_, washed in sterile PBS, and then fixed in 4% paraformaldehyde (PFA; catalog #P6148, Sigma-Aldrich) for 30 min. All cell culture experiments were run in duplicate within an experiment.

### RNA isolation and real-time quantitative RT-PCR (qPCR)

RNA from isolated microglia was extracted using the RNeasy Mini Kit (Qiagen; Song et al., 2012, 2014; Allender et al., 2018; Liu et al., 2018). Quantification of RNA was conducted using a NanoDrop Lite Spectrophotometer (Thermo Fisher Scientific). The expression of proinflammatory cytokine genes were each measured relative to GAPDH using TaqMan Assays-On-Demand primers (Thermo Fisher Scientific). Total RNA (1.5 μg) was used in a 20 μl reverse transcription synthesis reaction primed with Oligo-dT primers (Superscript First Strand Synthesis System, Thermo Fisher Scientific). Quantitative real-time PCR was performed in triplicate using 1× TaqMan Universal PCR master mix (Thermo Fisher Scientific) with the StepOnePlus Real-Time PCR System (Thermo Fisher Scientific) using the following primers and TaqMan probes: IL-1β, assay ID Mm00434228_m1; TNF-α, assay ID Mm00443258_m1; Nox2, assay ID Mm01287743_m1; and GAPDH, assay ID Mm99999915_g1 (Beers et al., 2011; Allender et al., 2018). Cycle threshold (Ct) values were calculated using monitor software (Opticon), with the threshold set at 40 SDs above background. The relative expression was calculated by normalizing the expression of individual genes to GAPDH and using the 2^−ΔΔCt^ method (Song et al., 2012, 2014; Allender et al., 2018; Liu et al., 2018).

### Intracerebroventricular cannula implantation

Intraventricular cannulas were implanted in 7- to 8-week-old 5XFAD mice using the experimental protocol listed on https://www.jax.org/∼/media/jaxweb/files/jax-mice-and-services/care-and-use-information.pdf?la=en (Liu et al., 2018). Each mouse was anesthetized with ketamine (150 mg/kg) and xylazine (10 mg/kg). Hair on the scalp was shaved with clippers, and the mice were placed into a stereotactic apparatus. A 2 cm midline incision starting at the base of the skull and directed anteriorly was fashioned using a #11 scalpel blade to expose the skull. A 1 mm hole was drilled 0.4 mm lateral and 1 mm posterior to bregma on the right side of the animal. The dura was nicked with a 26 gauge needle to ensure an egress of CSF and entrance into the subarachnoid space. A guide cannula (part #C315GS-2/SPC, Plastics One) was positioned in the hole and anchored into the skull using glass cement automix (part #1-GIG/ARFL, Instech Lab). The dummy and internal cannulas were used to seal the top of the guide cannula and infuse testing sample (parts #C315FDS-2/SPC, #C315IS-2/SPC, Plastics One). Animals were equally distributed into the experimental groups, taking into account gender, fur color, weight, and transgenic load after 710 d of postimplantation.

### Intracerebroventricular cannula injections

GlyB4 was prepared as described previously (Ma et al., 2009; Liu et al., 2018). The dosage was based on previously published observations (Calvo et al., 2010, 2011; Ma et al., 2011; Wang et al., 2015; Liu et al., 2018). To investigate whether exogenous NRG1 and GlyB4 can change microglial morphology and AD pathology, 5XFAD mice were divided into six groups and received intracerebroventricular injections of NRG1 or saline at 2–4 months of age (*N* = 8/group), GlyB4 or saline at 2–4 months of age (*n* = 21–26/group), or GlyB4 or saline at 4–6 months of age (*n* = 7/group), respectively. Briefly, recombinant human NRG1-β1-extracellular domain (catalog #377-HB, R&D Systems) was administered with a total of 5.4 ng in 5 μl PBS each week to produce a dose equivalent to 5 nm^17^ for 8 weeks (2–4 months of age). GlyB4 (4 μg in 5 μl), NRG1 (5 nm), or 5 μl of saline as a control was injected into 5XFAD mice for 8 weeks with implanted intracerebroventricular cannula under isoflurane anesthesia (5%; catalog #029405, Henry Schein Medical) weekly using a Hamilton syringe (catalog #80 262, Hamilton Robotics) at two different time points beginning at 8 or 16 weeks of age for 8 weeks total treatment time. All experiments described were conducted in strict accordance with good animal practice according to National Institutes of Health recommendations. All procedures for animal use were approved.

### Histopathology

The mouse brain was dissected after PBS perfusion (Liu et al., 2018), and the portion including hippocampus was sliced coronally on a mouse brain slicer according to the mouse brain atlas from Allen Institute. Tissue slices were fixed in 4% PFA (catalog #158127, Sigma-Aldrich) for 24 h, washed overnight in PBS, and immersed in 30% sucrose until saturated at 4°C. The tissues were embedded in Tissue-Tek O.C.T. Compound (Sakura Finetek USA), trimmed until the structure of the hippocampus was seen and 12 μm coronal sections were prepared and mounted on Superfrost Slides (Thermo Fisher Scientific).

### Immunofluorescence

Tissue staining for microglia, NRG1 receptor activation (phosphorylated erbB2), Aβ, and synapses was performed using the following antibodies specific for microglia: CD11b (1:200; rat anti-mouse monoclonal antibody; catalog #CBL1313, Millipore); Iba-1 (1:500; rabbit polyclonal; catalog #019–19741, Wako); phospho-erbB2/her2 (p-erbB2; Y1248; rabbit polyclonal, 15 μg/ml, catalog #AF1768, R&D Systems); MOAB-2 [mouse monoclonal antibody to Aβ peptide (Aβ40/42; 1:500; catalog #M1586-100, biosensis); and postsynaptic density-95 (PSD-95; 1:300; mouse monoclonal, clone k28/43; catalog #SKU75-028, MilliporeSigma, Davis, CA).

For MOAB-2, the slides were treated with 88% formic acid for 8 min before blocking. After nonspecific binding sites were blocked for 1 h at room temperature with blocking solution (5% normal goat serum, 5% BSA, and 0.05% Triton X-100 in TBS, pH 7.4), the primary antibody was diluted in the blocking solution and added onto the sections overnight at 4°C, followed by incubation with biotinylated anti-rat Ig (1:500; catalog #BA-9400, Vector Laboratories), streptavidin Alexa Fluor 488-conjugated (1:500; catalog #S11223, Thermo Fisher Scientific), Alexa Fluor 647-conjugated goat anti rabbit (1:500; catalog #A32733, Thermo Fisher Scientific), goat anti-rabbit Alexa Fluor 488 (1:500; Thermo Fisher Scientific), or goat anti-mouse Alexa Fluor 647 (1:500; catalog #A-21235, Thermo Fisher Scientific). The sections were counterstained with anti-fade medium with DAPI (catalog #P36962, Thermo Fisher Scientific) or neurotracer 530/615 (1: 500, catalog #N21482, Thermo Fisher Scientific) according to the manufacturer protocol, and mounted with mount medium (catalog #H-1200, Vector Laboratories).

### Quantitative image analysis

Microglia were imaged for quantification of bead phagocytosis using a CTR5500 scope (Leica), LS200US laser box (lasermet), EXi Aqua Camera (QImaging), and Surveyor imaging software. Images were acquired at 20× magnification. Internalization of beads within microglia was confirmed by confocal *z*-stack images taken using a confocal laser-scanning microscope (model LSM 710, Zeiss) with a Axio Imager Microscope Platform (Carl Zeiss Microscopy). Three images were acquired from each well in the eight-well chamber slide, corresponding to six images per condition within each independent experiment. This was also replicated in a second experiment, for a total of 12 images per condition. Images were uploaded to Fiji ImageJ (version 2.0.0-rc-65/1.52p) for analysis. Images were then coded and analyzed by an independent, blinded reviewer. The total number of GFP^+^ cells in each field was counted and within each cell, the number of 2 μm fluorescent beads was calculated. Cells approximating the edge of the image were not included in the analysis. The average number of beads per microglial cell is reported as well as a frequency histogram plotting the number of beads compared with the number of cells.

Image quantification was made between saline-treated and NRG1-treated or GlyB4-treated 5XFAD mice at the five regions of the brain, including the amygdala, thalamus, hippocampus, motor cortex, and somatosensory cortex. The brain coronal images (20×) were obtained with a fluorescence microscope (model DM5500B, Leica Microsystems) and scanned automatically with multiple channel options in a cooled CCD camera (model Q36526, QImaging). The images of different anatomic regions were taken with a laser-scanning confocal microscope (model LSM 710 Meta, Zeiss). The specific fluorescence-labeled structures on the images from both hemispheres were quantified for their pixel area and threshold percentage with MetaMorph Software version 7.8. The threshold value of each image was recorded in the original data.

The colocalization of microglia (CD11b) and NRG receptor activation (phosphorylated erbB2) was quantified using the Colocalization plugin on MetaMorph. Data are presented as a percentage of the colocalized phosphorylated erbB2 and CD11b.

In some of experiments, Iba-1^+^ microglia were classified as ramified, hyperramified, or clustered (Walker et al., 2013) according to their morphology and size (pixel area). In addition, to measure the extent of ramified microglial branches. We used the Sholl analysis from the Neuroanatomy package in ImageJ (version 1.54f; Young and Morrison, 2018). In brief, images were thresholded to select Iba1^+^ staining after the Unsharp Mask filter was applied. The Despeckle and Close tools were used to remove background staining and gaps between processes. Two cells per image were randomly selected from the images using a random coordinates generator. The Iba1^+^ cell closest to each random coordinate was chosen. In total, *n* = 20 microglia from six saline-treated AD mice and *n* = 18 microglia from four GlyB4-treated AD mice were used at the 4 month time point. At the 6 month time point, *n* = 32 microglia from five saline-treated AD mice and *n* = 50 microglia from five GlyB4-treated AD mice were analyzed. Each chosen microglial cell was cropped and skeletonized. The Sholl Analysis plugin was applied with a radius step size of 5 μm. The number of processes intersecting the concentric circles were tallied for each cell and plotted.

### Statistical analysis

Variables are presented as the mean and SE in microglia culture and *in vitro* phagocytosis assay. To compare bead phagocytosis across groups, one-way ANOVA with Tukey’s multiple-comparisons test was performed. A *p*-value of <0.05 was considered statistically significant for all analyses unless otherwise stated (*p < 0.05, **p < 0.01, ***p < 0.001). GraphPad Prism (version 9; GraphPad Software) was used to conduct the analysis.

Comparisons of tissue image quantification were made between saline-treated and NRG1-treated or GlyB4-treated 5XFAD mice at the five regions of the brain, including the amygdala, thalamus, hippocampus, motor cortex, and somatosensory cortex as well as the CA1 area. Groups were considered significantly different at *p* < 0.05 by a Student’s *t* test or two-way ANOVA.

Sholl data were analyzed using a two-way ANOVA to determine whether there is a significant main effect of treatment, distance from the soma, or treatment × distance interaction.

## Results

## NRG1 induces proinflammatory cytokines, promotes phagocytic activity and augments microglial activation and plaque formation in 5XFAD mice

**Figure 1.**
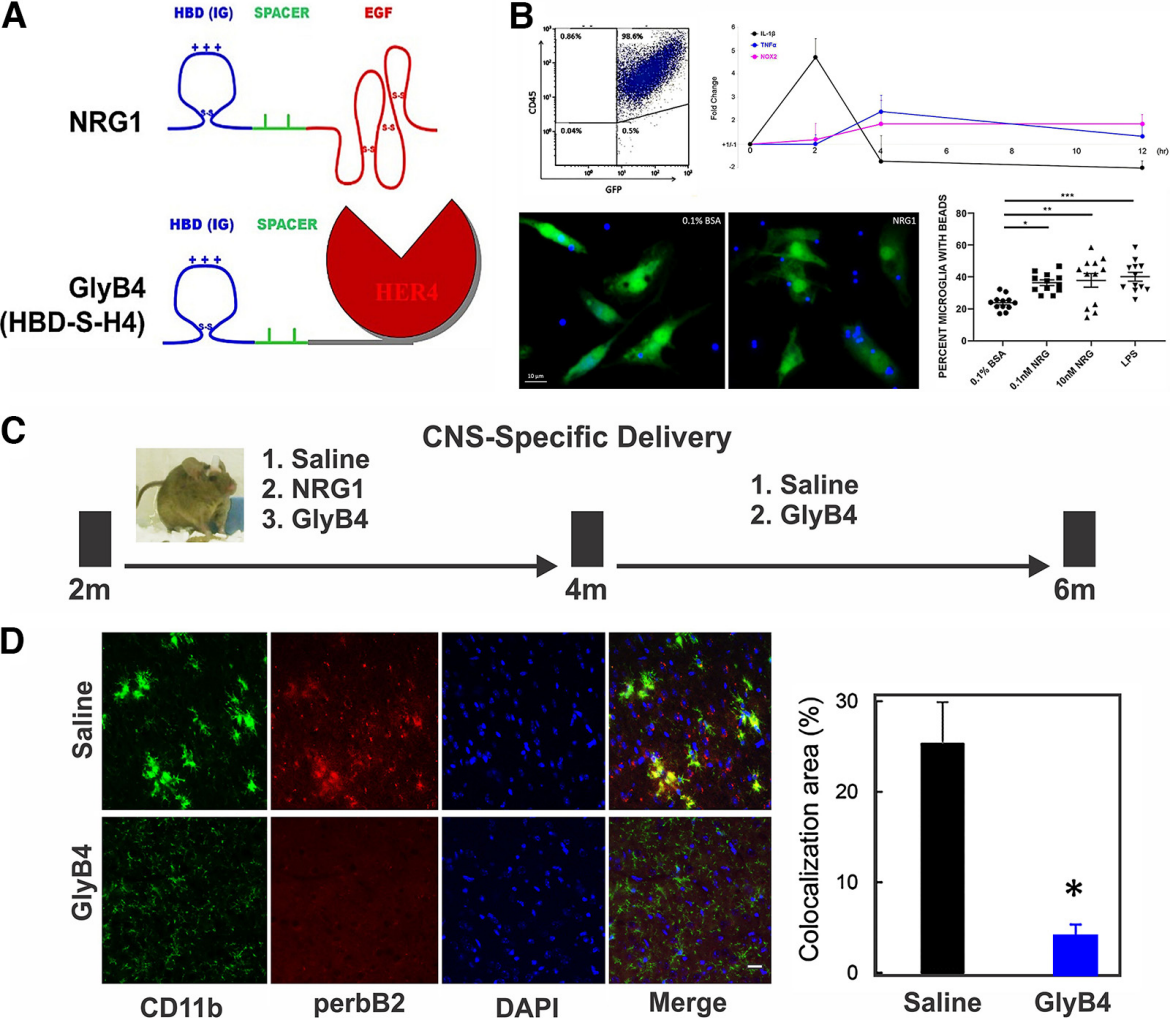
NRG1 induces proinflammatory cytokine expression and phagocytic activity in microglia that can be blocked *in vivo* by intraventricular GlyB4. ***A***, NRG1 contains a receptor-activating EGF-like domain linked to an Ig-like heparin-binding domain (HBD) that targets NRG1 within the CNS. GlyB4 (HBD-S-H4) is a NRG1 antagonist that fuses a HER4 NRG1 receptor with the HBD of NRG1 so that the antagonist is targeted to the same sites as NRG1. ***B***, Flow cytometry purified microglia (CX3CR1-GFP^+^CD45^+^) from CX3CR1-GFP mice (top left) were treated with 0.1 nm NRG1. Quantitative RT-PCR demonstrated increased proinflammatory cytokines IL-1β, TNF-α, and NOX2 peaking at different time points (top right; results expressed as the fold change of NRG1 vs control). Phagocytic activity of latex breads was markedly increased by NRG1 as measured by the percentage of microglia (green) containing latex beads (blue; bottom left panel). Scale bar, 10 μm. NRG1 had a dose-dependent effect compared with control (0.1% BSA) at 0.1 and 10 nm comparable to LPS (bottom right). The average number of beads per microglial cell is shown as the mean ± SEM *p < 0.05, **p < 0.01, ***p < 0.001. ***C***, Time course for the intracerebroventricular injection of saline, NRG1, or GlyB4 weekly into 5XFAD mice. ***D***, The 2-month-old 5XFAD mice treated weekly with GlyB4 or saline for 8 weeks (age, 2–4 months) showed reduced NRG1 receptor activation in microglia (CD11b; green) measured by phosphorylated-erbB2 (red) in the amygdala. Scale bar, 20 μm. *n* = 5/group. Quantitation expressed as the percentage of the colocalized area showed a more than fivefold reduction in NRG1 receptor activation with GlyB4 treatment (*p* < 0.05).

To elucidate the pathologic effects of NRG1 on microglia, we treated primary cultures of mouse microglial cells with a soluble, heparin-binding NRG1 isoform (Fig. 1*A*, top). Purified microglia from CX3CR1-GFP mice treated with NRG1-induced inflammatory cytokines including IL-1β, TNF-α, and NOX2, and promoted phagocytic activity as measured by latex bead uptake, similar to LPS stimulation in cultured mouse microglia (Fig. 1*B*).

**Figure 2.**
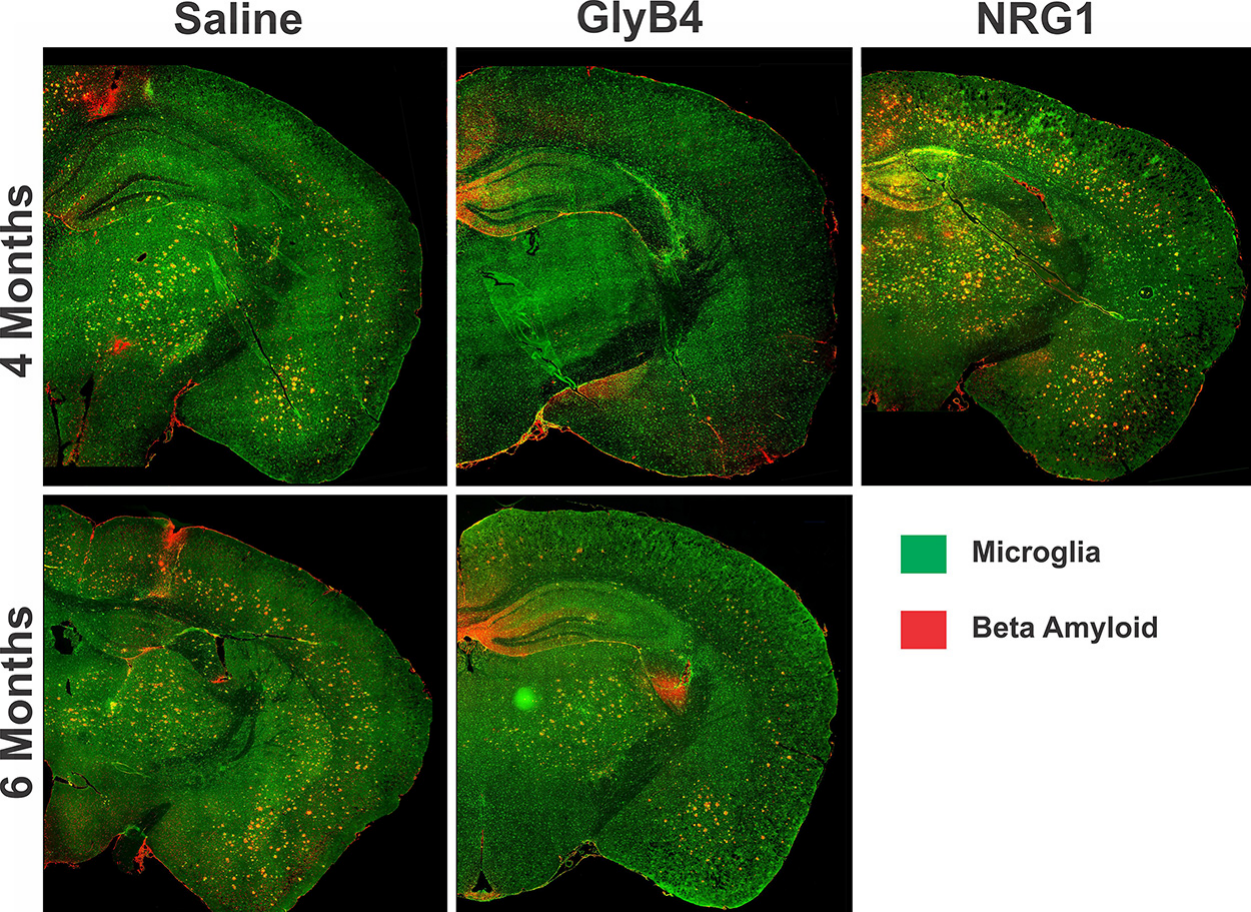
NRG1 treatment worsens AD pathology, while GlyB4 prevents microglial activation and Aβ plaque formation in 5XFAD mice. Mice were treated with saline, NRG1, or GlyB4 weekly for 8 weeks (2–4 months of age or 4–6 months of age) as outlined in Figure 1. Brains were stained for microglia (Iba1, green) and Aβ (MOAB2, red). Scale bar, 50 μm. Treatment between 2 and 4 months of age with NRG1 resulted in a marked increase in large, clustered microglia and Aβ plaques in the multiple brain areas, including the amygdala, thalamus, motor, and somatosensory cortex compared with saline-treated animals. In contrast, GlyB4 prevented the appearance of both the microglial clusters and Aβ plaques in all brain regions. By 6 months of age, the saline-treated animals showed a steady progression of histopathology, yet GlyB4 prevented new microglial activation and Aβ plaque formation that was comparable or less than that seen in saline controls at 4 months. At all of these time points, only the NRG1-treated animals showed any appreciable AD histopathology in the hippocampus at 4 months. Quantitative analysis of these pathologic findings are shown in Figure 3. Note: tissue wrinkles were digitally removed to improve visualization of the sections.

**Figure 3.**
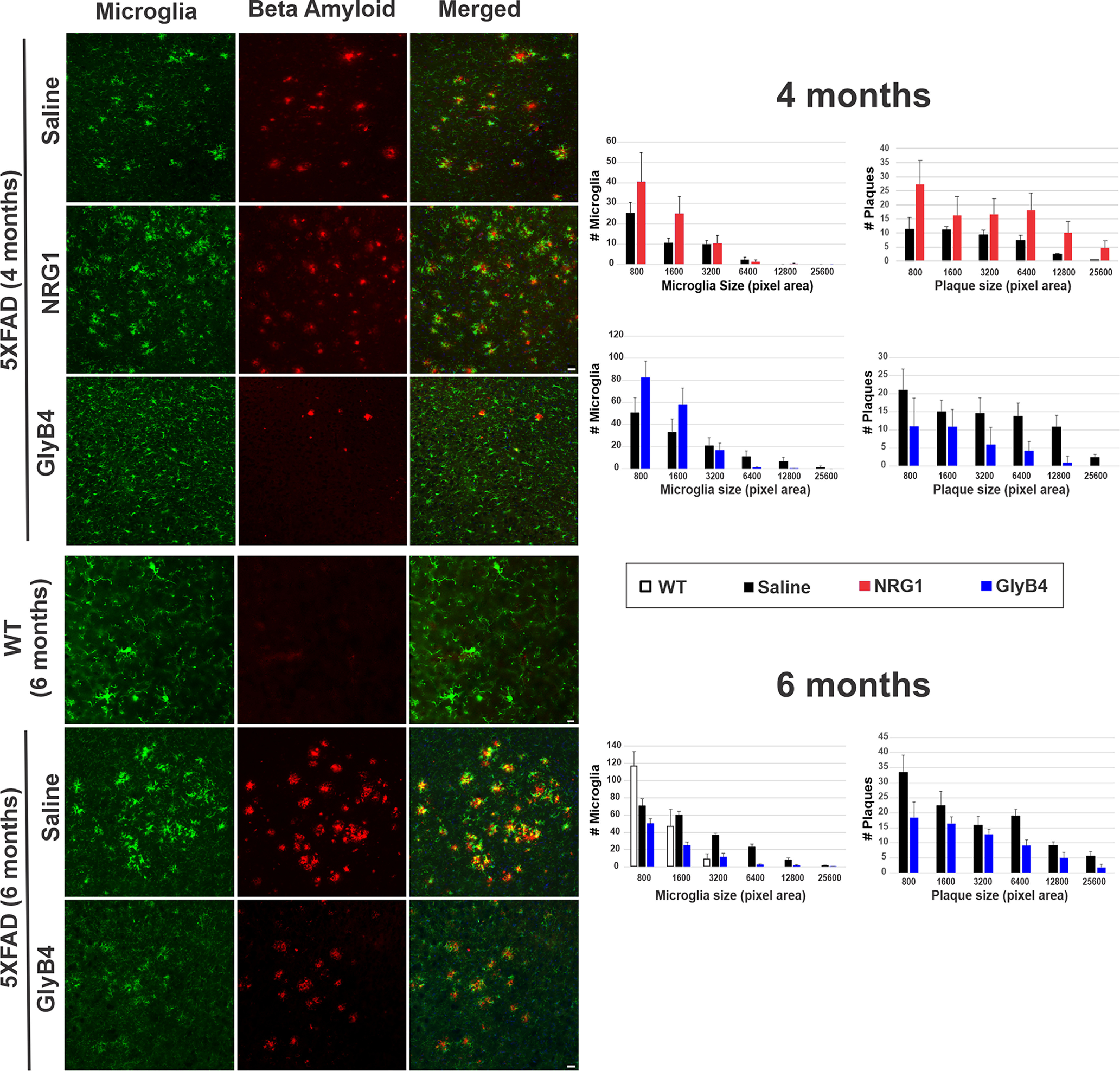
Quantitative changes in size and number of microglia and Aβ plaques after NRG1 and GlyB4 treatment. ***A***, Sections stained for microglial clusters (green) and Aβ plaques (red) as in Figure 2 from the amygdala show that NRG1 treatment results in increased clustered microglia (*p* = 0.06) and Aβ plaques (*p* < 0.05) compared with saline-treated 5XFAD mice. ***B***, ***C***, In contrast, at 4 months of age, GlyB4 prevented clustered microglia (*p* < 0.05) and Aβ plaque (6400 pixel area, *p < 0.05; 12,800 pixel area, *p < 0.05; 25,600 pixel area, *p < 0.05) formation in the amygdala; and at 6 months of age (6400 pixel area, **p < 0.01; ***B***) compared with saline-treated 4- and 6-month-old 5XFAD mice (***C***). Interestingly, NRG1 treatment increased the number of smaller individual cells and larger microglial clusters (as measured by pixel area/cell), while GlyB4 treatment at 4 months of age protected microglia staying in smaller sizes (800 pixel area, *p < 0.05) with ramified morphology, which shows a similar pattern to no-disease WT control mice. GlyB4 at 6 months of age decreased microglial clusters (800 pixel area, *p < 0.05; 1600 pixel area, ***p < 0.001; 3200 pixel area, **p < 0.01; 6400 pixel area, **p < 0.01). A summary of histologic changes in other brain regions is presented in Table 1. Scale bars, 50 μm.

In this study, we focused on early stages of disease in 5XFAD mice at 4 and 6 months of age. To determine whether these *in vitro* proinflammatory effects translate to neurodegeneration *in vivo*, NRG1 was injected directly into the CSF of 5XFAD mice through an intracerebroventricular (icv) cannula weekly for 8 weeks between 2 and 4 months of age (Fig. 1*C*). NRG1 significantly increased the development of large and clustered microglia and promoted Aβ plaque formation throughout the brain (Fig. 2). Brain areas affected included the amygdala, thalamus, and motor cortex (Fig. 3, Table 1).

**Table 1:** Brain region-specific changes in microglia and Aβ plaque density in 5XFAD mice treated with NRG1 or GlyB4

Treatment (time)	Pathology	Amygdala	Thalamus	Hippocampus	Motor cortex	Somatosensory cortex
NRG1 (2–4 m)	Microglia	2.6* (**↑**)	2.0* (**↑**)	0.9 (**–**)	2.6* (**↑**)	2.2* (**↑**)
	Aβ	2.3** (**↑**)	2.1*** (**↑**)	0.9 (**–**)	1.4* (**↑**)	0.7 (**–**)
GlyB4 (2–4 m)	Microglia	1.5* (**↑**)	0.4* (**↓**)	1.0 (**–**)	1.3 (**–**)	1.2 (**–**)
	Aβ	0.5** (**↓**)	0.6* (**↓**)	0.7 (**–**)	0.5* (↓)	0.7 (**–**)
GlyB4 (4–6 m)	Microglia	0.5* (**↓**)	0.6* (**↓**)	1.1 (**–**)	0.7* (**↓**)	0.6* (**↓**)
	Aβ	0.7* (**↓**)	0.4*** (**↓**)	1.4 (**–**)	0.5* (**↓**)	0.5** (**↓**)

Results are expressed as the ratio of either the NRG1-treated versus saline-treated mice or the GlyB4-treated versus saline-treated mice for the total number of microglia counts or Aβ plaque counts for the same time interval. *N* = 3 mice/group of NRG1-treated versus saline-treated mice (2**–**4 months of age); *N* = 5–8 mice/group of GlyB4-treated versus saline-treated mice (2**–**4 months); and *N* = 5–7 mice/group of GlyB4-treated versus saline-treated mice (4**–**6 months). *p < 0.05; **p < 0.01; ***p < 0.005 by Student’s *t* test. Arrows indicate significant up/down regulation or no change (**–**).

### Blocking NRG1 in microglia with GlyB4 prevents AD pathology in 5XFAD mice

GlyB4 was developed as a targeted NRG1 antagonist and has been used in studies to understand role of NRG1 in neural development (Ma et al., 2009, 2011). GlyB4 was designed to overcome a major challenge of drug delivery by concentrating the antagonist at the exact same sites where soluble heparin-binding forms of NRG1 act. This is achieved by fusing a soluble “decoy” HER4 NRG1 receptor antagonist with the heparin-binding domain of NRG1, as shown in the bottom of Figure 1*A* (Ma et al., 2009). GlyB4 has been successfully used *in vitro* and *in vivo* to block NRG1 actions in studies on neural development and models of other diseases (Calvo et al., 2010, 2011; Ma et al., 2011; Liu et al., 2018).

As a means of demonstrating target engagement, Figure 1*D* shows that GlyB4 injected into the brains of 5XFAD mice through an intracerebroventricular catheter over 8 weeks between 2 and 4 months of age effectively blocked NRG1 receptor activation on microglia as measured by NRG1 erbB2 receptor phosphorylation. Opposite to the proinflammatory and neurodegenerative effects produced by NRG1, GlyB4 prevented microglial activation and clustering and Aβ plaque formation throughout the brain (Fig. 2). Quantitative reductions were seen in brain areas including the amygdala, thalamus, and motor cortex (Fig. 3, Table 1).

In 5XFAD animals, microglial clusters, many with amoeboid shapes, appear closely localized with Aβ plaques (Figs. 2, 3). Each cluster contains three to four microglia with multiple DAPI-stained nuclei. In contrast, WT control mice have only small ramified microglia without clustering or amoeboid shapes (Fig. 3). Adding additional intraventricular NRG1 from 2 to 4 months of age significantly increased larger microglia clusters (*p* = 0.06) and Aβ plaque formation (*p* < 0.01; Fig. 3, Table 1). Consistently, blocking NRG1 with GlyB4 prevented both plaque formation and microglial clustering. From 2 to 4 months of age, GlyB4 treatment led to increased smaller, more ramified microglia.

### At later stages of disease GlyB4 prevents new microglial activation, plaque formation, and synaptic loss

Given the ability of GlyB4 to prevent early microglial activation and plaque formation before the onset of significant histopathology, we determined the effects of GlyB4 once the histopathology has begun at 6 months of age in 5XFAD mice (Fig. 1*C*). Blocking NRG1 with GlyB4 for 8 weeks between 4 and 6 months of age did not reduce existing histopathology (Fig. 2). Instead, it prevented further increases in microglial clustering and plaque formation throughout the brain. This was seen in the amygdala, thalamus, motor cortex, and somatosensory cortex (Fig. 3, Table 1). While NRG1 increased large (pathogenic) microglial clusters, GlyB4 treatment prevented these microglial clusters from forming at both early and later time points. We also found that astrogliosis was also reduced at this later stage with GlyB4 treatment in 5XFAD mice (data not shown). This could be an indirect effect, similar to that observed with GlyB4 treatment of the ALS SOD1 model (Liu et al., 2018).

**Figure 4.**
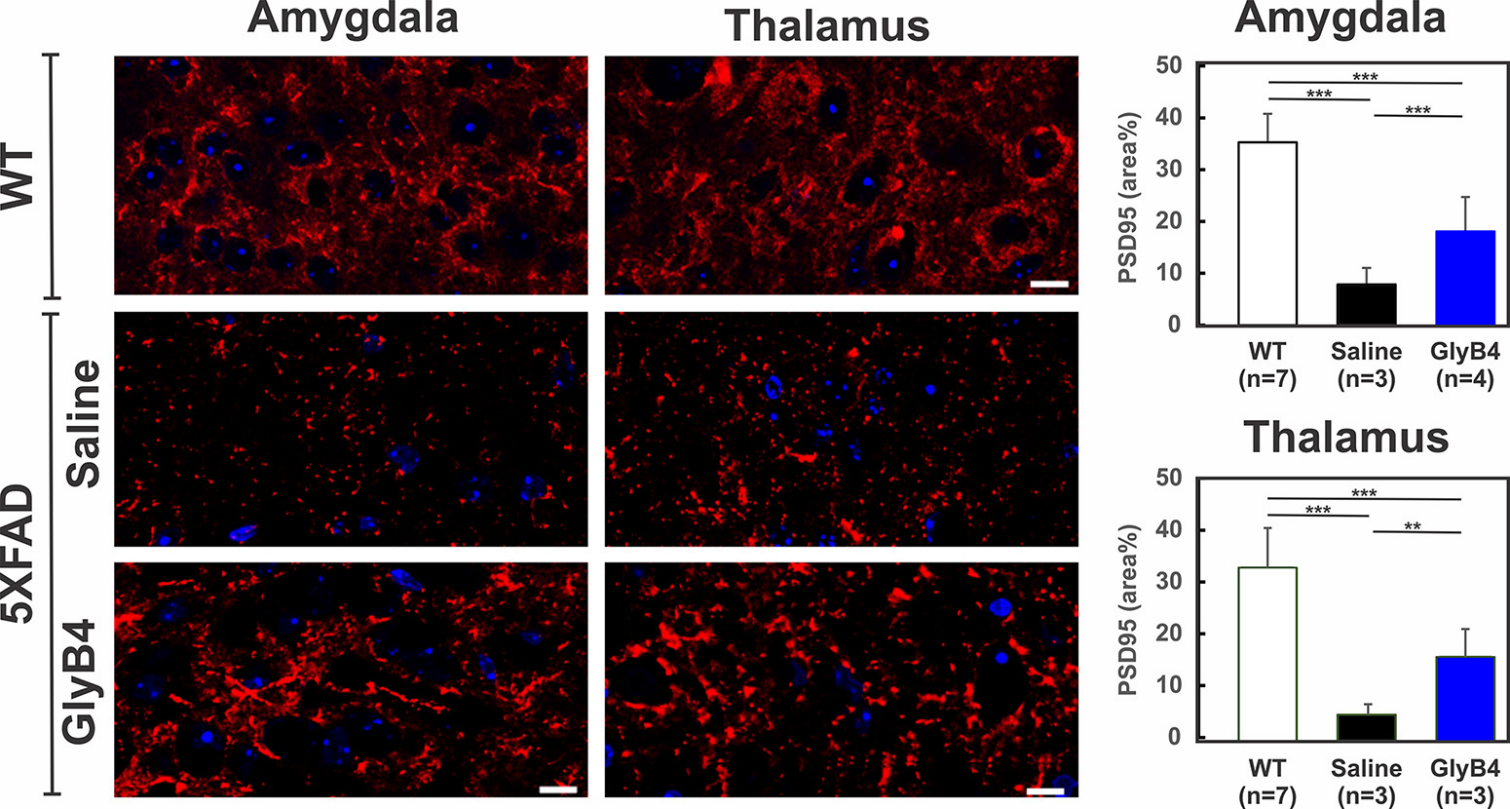
GlyB4 prevents excitatory synaptic loss in AD mice. As a means to understand the downstream effects of GlyB4 treatment on neuronal function, we quantified excitatory postsynaptic synapses using PSD-95 (red) and DAPI (blue). Confocal microscopy and quantitative analysis of the amygdala and thalamus demonstrated that GlyB4 treatment rescues excitatory synaptic loss compared with saline-treated 5XFAD mice as well as WT mice. Scale bar 10 μm. *p < 0.05, **p < 0.01, ***p < 0.001.

While it is not feasible to do behavioral studies at early stages of the 5XFAD mouse model (<6 months of age), as a means to assess the functional consequence of blocking AD pathology we measured synaptic density as a surrogate marker for cognitive behavior. In fact, synaptic loss is the strongest correlate of cognitive decline in AD and is likely mediated through microglial activation with the most synaptic loss occurring in the hippocampus after 9 months of age (Rajendran and Paolicelli, 2018). We quantified excitatory synaptic density in multiple brain regions using PSD-95 in multiple brain regions. Not surprisingly, the greatest degree of synaptic loss was present in regions of maximal microglial activation and Aβ deposition, as shown in Figure 4 for PSD-95 in the amygdala and thalamus. GlyB4 treatment from 4 to 6 months significantly reduced PSD-95 synaptic loss compared with saline-treated animals, but did completely rescue them compared with WT controls (Fig. 4). This result is similar to previous findings that GlyB4 reduces both motor neuron and synaptic loss in the SOD1 ALS model (Liu et al., 2018; J. Liu, J.A. Loeb and F. Song unpublished manuscript). We found similar results with additional synaptic markers including synaptophysin (data not shown).

### GlyB4 changes microglia to a more ramified but less pathogenic morphology

**Figure 5.**
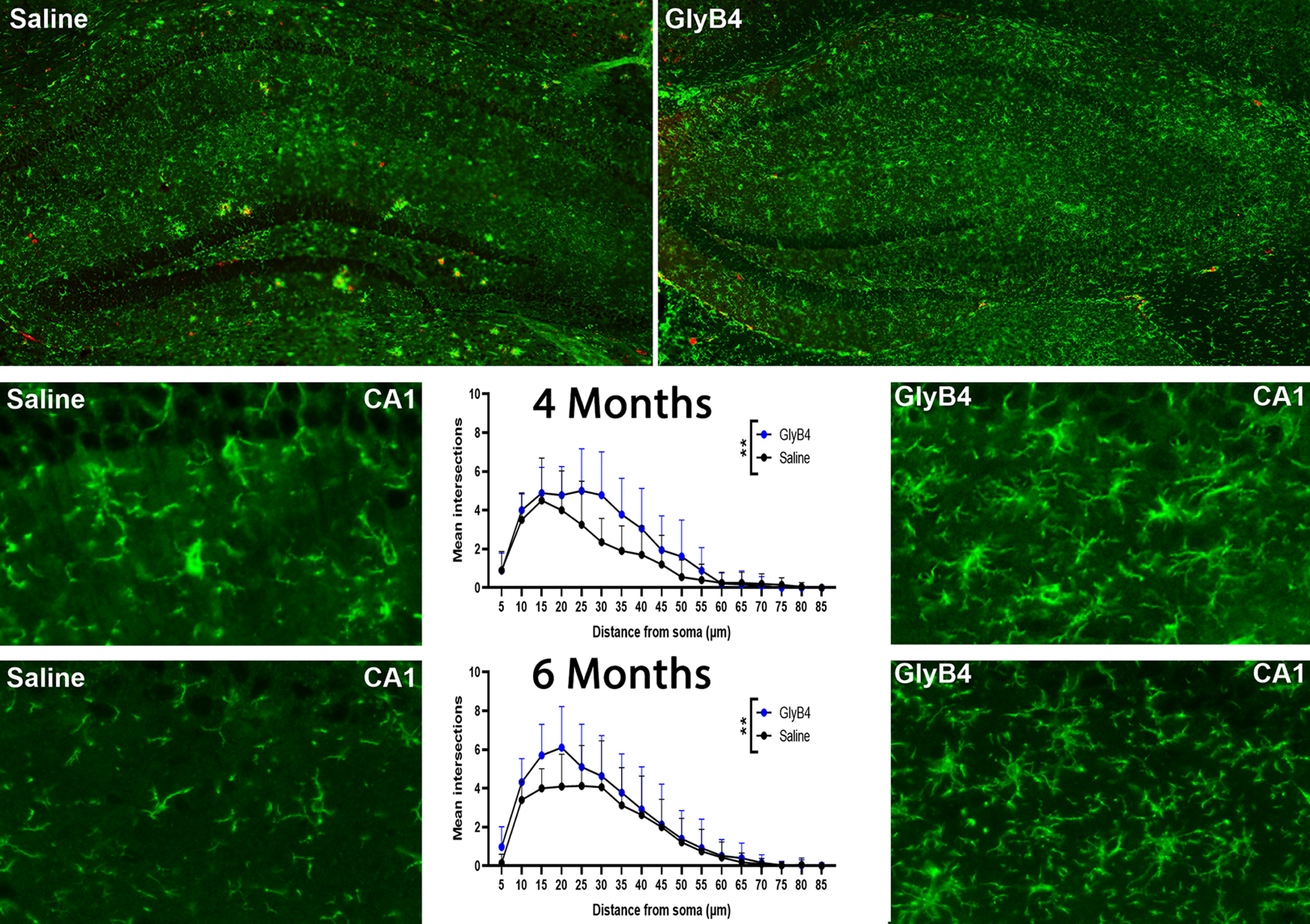
GlyB4 treatment produces phenotypic changes in microglial morphology in the hippocampus before plaque formation. As a means to understand the effects of GlyB4 on microglia in a brain region before significant Aβ plaque formation, we quantified microglial numbers and morphology at 4 and 6 months compared with saline-treated controls using a Sholl analysis. At 6 months of age (top panels) GlyB4 treatment led to marked changes in both microglial numbers (Iba1, green) and morphology. Scale bar, 100 μm. Quantitative analysis of the CA1 region showed that this is because of an increase of more branches that intersect with Sholl analysis circles compared with saline-treated AD mice at both 4 months (**p < 0.005) and 6 months (**p < 0.005). Additionally, a significant effect of distance (****p < 0.0001) and a treatment × distance interaction (****p < 0.0001) were observed at both 4 and 6 months of age. Scale bar, 20 μm.

While the hippocampus is one of the earliest affected brain regions in AD patients and its dysfunction is believed to underlie the core feature of memory impairment (Maruszak and Thuret, 2014), in the 5XFAD mouse model microglial clustering and Aβ plaque formation occur much later in the hippocampus than in other brain regions (Table 1). We took advantage of this to assess the effects of GlyB4 on microglia before the onset of Aβ plaque deposition. At both 4 and 6 months of age where microglial clusters have not been seen, we examined the morphology of microglia in the CA1 hippocampus region in 5XFAD mice. On gross inspection, GlyB4 changed microglial morphology from small quiescent forms to larger, hyperramified shapes at both time points (Fig. 5). We next used a Sholl analysis to quantify the number of processes and their distance from the cell). These findings combined with similar results obtained using GlyB4 in the SOD1 ALS model (Liu et al., 2018) suggests that while NRG1 signaling produces a significant clustering morphology (a pathologic phenotype), blocking NRG1 before the onset of plaque deposition leads to changes in microglia morphology and activity “upstream” from the neurodegenerative process that transforms microglial into a less pathogenic phenotype.

## Discussion

Therapeutics that target by-products of the neurodegenerative process, such as Aβ and tau, have not effectively translated to highly beneficial treatments in humans and therefore alternative therapeutic targets are badly needed (Kantarci, 2022). Common to all neurodegenerative diseases is progressive neuroinflammation associated with synaptic and neuronal loss leading to clinically important neurologic dysfunction. In AD, neuroinflammation is mediated by microglial cells and has been demonstrated in genetic, preclinical, and clinical studies (Malik et al., 2015; Ardura-Fabregat et al., 2017). Growing evidence suggests that microglial phagocytosis could be a key player in the synaptic loss in amyloid mouse models. One hypothesis suggests that microglial activation results from amyloid deposition and is beneficial for plaque removal (Daria et al., 2017). Others suggest that chronic exposure to amyloid may become toxic to microglia preventing them from phagocytosing toxic debris (Mandrekar-Colucci and Landreth, 2010; Wendt et al., 2017). However, similar to our findings here, microglial activation has been observed before plaque-forming stages of the disease in animal models (Hanzel et al., 2014) and in neuroimaging studies in patients with MCI before evidence of amyloid deposition (Okello et al., 2009; Hamelin et al., 2016), suggesting that neuroinflammation is an early or upstream event in AD and therefore a viable therapeutic target.

Here we show that targeting NRG1-induced neuroinflammation through selective blockade of NRG1 signaling in microglia can prevent as well as stop existing disease in an AD mouse model. Consistently, this treatment prevented histologic changes in inflammation, plaque formation, and synaptic loss throughout the brain. While NRG1 is a critical glial growth and differentiation factor during development, in the adult NRG1 provides highly localized signaling between neurons and surrounding microglia. In addition to the findings presented here for AD, NRG1 signaling has been shown to be pathogenic in models of chronic pain (Calvo et al., 2010, 2011), multiple sclerosis (Allender et al., 2018), and ALS (Song et al., 2012, 2014; Liu et al., 2018; Schram et al., 2020). In all of these models, blocking NRG1 in microglia does not eliminate microglia, but instead changes their phenotype to one that is no longer pathogenic. Phenotypic changes in microglia, including alterations in morphology, proteomic signatures, and behaviors, are associated with disease progression (Nguyen et al., 2017; Yin et al., 2017). Consistently, we found that GlyB4 treatment not only leads to reduced large microglial clusters associated with plaques, but also promotes more highly ramified microglia compared with normal controls that appear to be less pathogenic.

Given the diverse actions of NRG1 from alternatively spliced isoforms, it is not surprising that forms of NRG1 have also been reported to provide modest beneficial effects after the onset of cognitive impairments in AD mouse models (Ryu et al., 2016; Xu et al., 2016; Ou et al., 2021). Ryu et al. (2016) reported that intracerebroventricular infusion of a fragment of the NRG1 protein attenuated cognitive impairments in 13-month-old Tg2576 AD mice (Ryu et al., 2016). Xu et al. (2016) showed that overexpressing membrane-bound forms of NRG1 using a lentiviral vector directly into the hippocampus produced modest improvements in cognition compared with the lentiviral vector alone and in a 9-month-old AD line (Xu et al., 2016). While Ryu et al. (2016) infused a small 7 kDa peptide consisting of only the EGF-like domain that lacks the heparin-binding targeting domain (Fig. 1*A*), Xu et al. (2016) used transmembrane precursors of type I and III NRG1 (Xu et al., 2016). The specific splice form of NRG1 is critical in how and where NRG1 is targeted.

Exactly how NRG1 promotes neuroinflammation and downstream neuropathology is not entirely clear; however, previous mechanistic studies have shown a direct effect of NRG1 on microglia mediated through erbB2-4 receptors (Calvo et al., 2010, 2011) and the MEK/ERK1/2 pathway (Calvo et al., 2011). Specifically, NRG1 released into the dorsal horn of the spinal cord from dorsal root ganglia sensory neurons stimulates proliferation, survival, and chemotaxis of microglia that can be inhibited with NRG1 inhibitors, including GlyB4 (Calvo et al., 2010, 2011). Transcriptomic analyses of microglial subtypes compared with known transcriptomic subtypes would be important next steps.

GlyB4 is a full-length secreted NRG1 protein containing both the EGF-like domain and heparin-binding domain needed for selective microglial targeting (Ma et al., 2009; Liu et al., 2018; Loeb and Song, 2019). GlyB4 takes advantage of this same heparin-binding domain targeting system. We combined this with CNS-specific delivery through an intracerebroventricular catheter and showed that GlyB4 can selectively prevent the activation of NRG1 receptors on microglia. While direct delivery of GlyB4 into the CSF could be seen as a disadvantage for downstream clinical treatment, it has the key advantages of enhancing brain efficacy and reducing peripheral side effects. Intrathecal delivery of drugs has become a more accepted approach for CNS-specific drug delivery for many other brain disorders (Calvo et al., 2010, 2011; Allender et al., 2018; Liu et al., 2018). Once in the CSF, GlyB4 exerts its effects evenly throughout the brain and spinal cord (Calvo et al., 2010; Liu et al., 2018), suggesting that the heparin-binding domain enables GlyB4 to penetrate deep within the CNS. Most biologics, including antibodies, have very poor CNS penetration. The novel technology used to fuse the heparin-binding domain of NRG1 to HER4, therefore, could also be used to generate other targeted drugs with improved brain penetration (Freskgard and Urich, 2017).

## Synthesis

Reviewing Editor: Harald Sontheimer, University of Virginia Decisions are customarily a result of the Reviewing Editor and the peer reviewers coming together and discussing their recommendations until a consensus is reached. When revisions are invited, a fact-based synthesis statement explaining their decision and outlining what is needed to prepare a revision will be listed below. The following reviewer(s) agreed to reveal their identity: NONE. Note: If this manuscript was transferred from JNeurosci and a decision was made to accept the manuscript without peer review, a brief statement to this effect will instead be what is listed below.

This is a transferred paper with prior reviews from JNS. The authors have addressed the previous critiques to my satisfaction. This is an intereseting and novel study.
